# Combined and Hybrid Treatments of Hyaluronic Acid (HA) and Calcium Hydroxylapatite (CaHA): A Systematic Review of Mechanisms of Action, Aesthetic Effectiveness, Satisfaction, and Safety Profile

**DOI:** 10.1007/s00266-025-04904-x

**Published:** 2025-06-06

**Authors:** Renald Meçani, Mojgan Amiri, Jonathan Kadouch, Dusan Sajic, Frank Lin, Jessie Cheung, Diana Barrera, Omar Haroon, Susana Sil-Zavaleta, Yates Chao, Taulant Muka

**Affiliations:** 1 Epistudia, Bern, Switzerland; 2 Department of Endocrinology and Diabetology, Medical University of Graz, Graz, Austria; 3 Practice for Aesthetic Dermatology, ReSculpt Clinic, Amsterdam, The Netherlands; 4 Guelph Dermatology Research, Guelph, ON Canada; 5 Faculty of Health Sciences, McMaster University, Waterloo, ON Canada; 6 Derma Skin Institute, Guelph, ON Canada; 7 Department of Medicine, Nursing and Health Sciences, Monash University, Clayton, Victoria Australia; 8 Cheung Aesthetics & Wellness, Willowbrook, IL USA; 9 Universidad de la Sabana, Chía, Colombia; 10 Hospital de San José, Bogotá, Colombia; 11 Fundación Universitaria de Ciencias de la Salud, Bogotá, Colombia; 12 Department of Hand and Plastic Surgery, Thurgau Hospital Group, Frauenfeld, Switzerland; 13 Universidad Anahuac, México City, Mexico; 14 Hospital Ángeles del Pedregal, México City, Mexico; 15 Chao Institute of Dermatology, Taipei, Taiwan

**Keywords:** Hyaluronic acid, Calcium hydroxylapatite, Fillers, Dermatology, Cosmetic, Aesthetics

## Abstract

**Background:**

The use of dermal fillers has become increasingly popular in aesthetic medicine for facial rejuvenation and skin enhancement. Hyaluronic acid (HA) and calcium hydroxylapatite (CaHA) are particularly well-known for their unique benefits. This systematic review evaluates the combinations (e.g., using two separate treatments together) and hybrid treatments of HA and CaHA (e.g., direct syringe-to-syringe mixing), focusing on their mechanisms of action, aesthetic effectiveness, patient satisfaction, and safety.

**Methods:**

Six bibliographic databases were searched for human and non-human studies that explored the mechanistic effects of combined and hybrid HA and CaHA treatments, and their aesthetic effectiveness, skin quality, satisfaction, and safety were included. Due to high heterogeneity among included studies, a qualitative synthesis of the evidence was performed.

**Results:**

The combination of HA and CaHA stimulates collagen synthesis through different molecular pathways. The combined and hybrid treatments are associated with high aesthetic effectiveness in various facial areas, as reported by both investigators and subjects. Additionally, the combinations and hybrid treatments show high satisfaction rates and have a safe profile, with minor, self-resolving adverse effects. A trend of slight decline in both aesthetic improvement and treatment satisfaction was observed after six months.

**Conclusions:**

The combinations and hybrid treatments of HA and CaHA appear to be a promising, safe, and efficacious treatment for facial rejuvenation, offering both immediate volumizing effects and longer-term benefits through collagen synthesis. Future research should employ rigorous study designs aimed at refining treatment protocols and generating strong, long-term evidence for the safety and effectiveness of this combination or hybrid therapy in aesthetic medicine.

**Level of Evidence II:**

This journal requires that authors assign a level of evidence to each article. For a full description of these Evidence-Based Medicine ratings, please refer to the Table of Contents or the online Instructions to Authors www.springer.com/00266.

**Supplementary Information:**

The online version contains supplementary material available at 10.1007/s00266-025-04904-x.

## Introduction

The use of dermal fillers in aesthetic medicine has become increasingly popular for addressing facial rejuvenation and enhancing skin quality [1]. Among the various fillers available, hyaluronic acid (HA) and calcium hydroxylapatite (CaHA) are two well-known substances, each offering distinct benefits [2–4]. HA is renowned for its hydrating and volumizing properties, while CaHA is recognized for stimulating collagen production and providing longer-lasting structural support [4–6]. Given their complementary mechanisms of action, the combined use of HA and CaHA may influence aesthetic outcomes [7–9].

Products containing both substances have been approved for use in multiple regions globally [10]. In addition, physicians have been practicing the off-label injection of mixtures of HA and CaHA products available on the market. This practice is marked by significant heterogeneity in administration protocols and a lack of standardization in terms of products and ratios used [8, 11, 12], leading to uncertainty regarding treatment efficacy and safety. Various studies exploring the efficacy of combining both HA and CaHa have been conducted, with some studies showing promising results and a safe profile [13–15]. Nevertheless, to date, there is no comprehensive review of the evidence on the efficacy and safety of this practice.

This systematic review evaluates the combination and hybrid treatments of HA and CaHA, focusing on their mechanisms of action, aesthetic effectiveness, patient satisfaction, and safety. By examining studies involving both human and non-human subjects, it aims to bridge current knowledge gaps, provide practical insights for practitioners, and clarify the clinical implications of these findings. In doing so, it also highlights areas where further research is needed.

## Methods

### Review Design

This study was conducted based on recent systematic review guidelines and reported following the PRISMA reporting standards [16–18]. The study protocol of this project was registered in the OSF Registries (doi.org/10.17605/OSF.IO/6VBK2).

### Data Sources and Search Strategy

The databases Embase.com, Medline ALL (Ovid), Web of Science Core Collection, and Cochrane Central were searched from their inception until April 11, 2024. Additionally, the first 300 results from Google Scholar were included. A search strategy was developed by an expert librarian, focusing on terms related to hyaluronic acid (HA) and calcium hydroxylapatite (CaHA). The specifics of the search strategy and keywords are detailed in Table S1. To find further relevant studies, the reference lists of the final included studies and available relevant systematic reviews were manually reviewed.

### Inclusion and Exclusion Criteria

The inclusion criteria were as follows: published human studies (interventional and observational, participants aged  ≥ 18) or non-human studies (in vitro and animal studies) investigating the combinations (e.g., using two separate treatments together) and hybrid treatments of HA and CaHA (e.g., direct syringe-to-syringe mixing). The treatments could be administered as a premixed product, immediately after each other, or with a time interval. Studies needed to assess outcomes related to mechanism of action, aesthetic effectiveness, skin quality parameters, satisfaction, and safety, regardless of the type of injected hyaluronic acid, participants' characteristics, health status, treated regions, or sample size. Reviews, letters to editors, and conference abstracts were excluded.

### Study Selection and Data Extraction

Two reviewers independently evaluated titles and abstracts of the retrieved references and subsequently assessed the selected full texts for eligibility. Data from the eligible studies were extracted using a predefined form, which included details such as the first author’s name, study design, publication year, location, number of participants, sex distribution, part

icipants’ health status or characteristics at the start of the study, age, follow-up duration, ethnicity, skin type, treated area, injection ratio of mixed products, injection order, injection interval of the fillers, injection protocol, dilution and dosage, name and brand of dermal fillers, outcome assessment methods, adjustments, and measures of frequency or association.

### Quality Assessment and Statistical Analysis

Risk of bias in the included studies was evaluated using different tools depending on the study design and whether the study involved humans or non-humans. For non-randomized studies, such as observational and pre-post interventional studies, the Risk Of Bias In Non-randomized Studies of Interventions (ROBINS-I) tool was utilized [19] and randomized controlled trials were evaluated with the Cochrane Collaboration’s Tool Risk of Bias 2 (ROB2) [20]. In vitro studies were assessed using the Quality Assessment Tool for In Vitro Studies (QUIN Tool) [21], and animal studies underwent evaluation using The Systematic Review Centre for Laboratory Animal Experimentation Risk of Bias tool (SYRCLE) [22].

### Statistical Analysis

Given the limited number of studies and the heterogeneity in study design and measurement tools, we conducted a qualitative review. The characteristics and findings of the included studies are summarized in separate tables.

## Results

### Study Selection

**Fig. 1:**
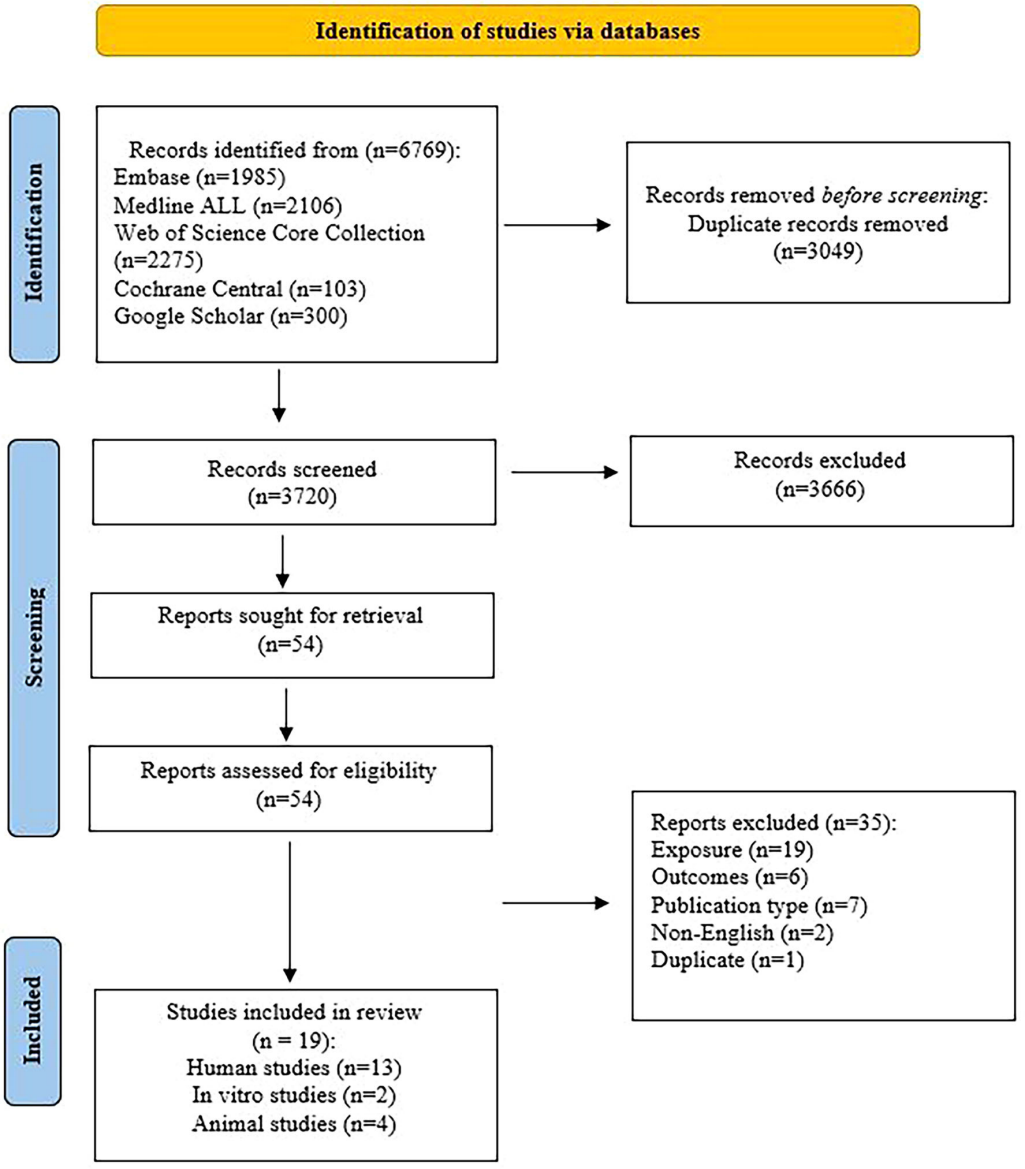
Flowchart of identification, screening, eligibility, inclusion, and exclusion of retrieved studies

Out of 3720 references, 54 full texts were reviewed for potential inclusion. Of these, 35 studies were excluded for various reasons: inappropriate exposure (other treatments were applied or the interventions of interest were used), irrelevant outcomes (focused on aspects such as physiochemical properties of fillers rather than aesthetics, mechanism of action, satisfaction, and safety), unsuitable publication types (such as abstracts, perspectives, letters to editors, and reviews), and non-English papers. Nineteen studies were included in the current review, comprising thirteen human studies, two in vitro, and four animal studies. Figure 1 represents the study selection procedure.

### Study Characteristics

Among thirteen human studies, the median number of participants was 39 (Interquartile range (IQR): 15-70) with a median age of 48.7 years (IQR: 45.9-56.7) and a median follow-up duration of 7.5 months (IQR: 5.5-12). Eight studies were interventional (five pre-post and three controlled trials), five studies were observational (four retrospective and one prospective), and one was a case report study. One retrospective observational study did not report on follow-up duration. Six studies (46.1%) included only female participants, while the remaining studies included both female and male participants, with over 88% of the total participants being female. Out of ten monocentric studies, three of them were performed in Italy, two studies in the USA, two studies in Brazil, one in South Korea, one in the Russian Federation, and one in Chile. Out of three multicentric studies, two of them were performed in The Netherlands and Lebanon, one in Spain, Brazil, and Italy. In addition, four animal studies conducted on mice and two in vitro studies on fibroblasts and human keratocytes were included. Out of the studies who reported CaHA/HA mixing ratios, two studies used 1:1 to 1:3 for slight corrections, for mild corrections and for severe correction, and six studies used premixed products with the manufacturer’s ratio. Regarding the injection order, the majority of the studies (*n *= 10) reported a concomitant injection, while fewer studies (*n *= 3) reported on injections given separately in time (Tables 1 and 2).

**Table 1:** *C*haracteristics of the human studies

Author, year	Country	Design	Follow-up	No. participants (Int/Cnt)^1^	Sex (%F)	Health status/characteristics	Age^2^	Ethnicity
Kadouch [11]	Netherlands, Lebanon	Observational, Retrospective (Patients records)	12 m	134	B (95)	Healthy adults	43 (18-78)	NR
Fakih-Gomez [8]	Netherlands, Lebanon	Observational, Retrospective (Chart review)	12 m	41	F	Healthy adults	47.5 (21-63)	NR
Bravo [7]	Brazil	Interventional, Pre-post	120d	15	B (93.3)	Healthy adults with mild and moderate sagging of the jawline	45.9±9	NR
Chang [29]	South Korea	Interventional, Pre-post	9m	25	B (88)	With 1 or 2 score on the validated Merz 5-point scale for nasolabial fold and jawline contour at rest	48.7 (36-68)	NR
		Interventional, Controlled (Histological results)	6m	5 (4/1)	NR	With 1 or 2 score on the validated Merz 5-point scale for nasolabial fold and jawline contour at rest	NR	NR
Urdiales-Galvez [27]	Spain, Brazil, Italy	Interventional, Pre-post	180d	15	F	Healthy women who had undergone previous minimally invasive aesthetic procedures	58 (51-62)	NR
Barone [13]	Italy	Interventional, Controlled	12m	65 (22/43) [*Cnt1*: 20; *Cnt 2*: 23]	B (94.7)	Who had primary surgical or non-surgical treatment for elastosis	62 (50-80)	NR
Wortsman [30]	Chile, Brazil, Netherlands	Observational, Retrospective	NR	21	B (90)	Patients ≥18 years with confirmed injection of CaHA clinically and ultrasonographically	52±12.8	NR
Godin [15]	USA	Observational, Retrospective	*Int:* 11.5m *Cnt:* 15m	61 (15/46)	B (NR)	NR	*Int*: 56 (38-78) *Cnt*: 54 (31-78)	NR
Ilaria [33]	Italy	Case-report	12m	1	F	With asymmetrical facial lipoatrophy	40	NR
Yutskovskaya [28]	Russian Federation	Interventional, Controlled	5m	8 (4/4)	F	Healthy women	35-45	NR
Zerbinati [32]	Italy	Observational, Prospective	24w	60	B (93.3)	With moderate to severe age-related mid-face volume deficit	56.7 (26-70)	NR
Somenek [12]	USA	Interventional, Pre-post	6m	12	F	With moderate-to-severe wrinkles in the perioral region	62.5 (51-70)	NR
Felix Bravo [9]	Brazil	Interventional, Pre-post	120d	15	F	With facial skin laxity	41.5 ± 2.9	NR

Author, year	Treated sites	Injection order	Product names	Mixing ratio (CaHa: HA)	Volume injected	Treatment protocol/number of sessions	Outcomes
Kadouch [11]	Full face (including cheeks, jawline, nasolabial folds, marionette lines, parotid area, chin, and temples; anterior and/or posterior cheeks; jawline and cheeks; anterior and/or posterior jaw); and neck.	At the same time, mixed manually before injection	HA (Belotero Balance or Volume, Merz Pharmaceutical GmbH). CaHA (Merz Pharmaceutical GmbH).	1:1 to 1:3 for slight correction, 1:4 to 2:4 for mild correction, 2:6 to 3:8 for severe correction	Midface zygoma and cheeks: 2.6 cc (1.3 cc/side). jawline and cheeks: 2.6 cc (1.3 cc/side). combination of facial areas: 4.5 to 15 cc (split across both sides of the face). [Varied depend on the number of areas being treated and individual patient characteristics]	Subcutaneous injections using a fanning technique with a 25G, 38- or 50-mm cannula with fanning injection technique Single session	Safety
Fakih-Gomez [8]	Midface and lower face	At the same time, mixed manually before injection	HA (Belotero Volume, Merz Pharmaceuticals). CaHA (Radiesse, Merz Pharmaceuticals)	1:1 to 1:3 for slight correction, 1:4 to 2:4 for mild correction 2:6 to 3:8 for severe correction	volumes ranged from 3.5 to 15.0 cc (mean±SD: mean = 9.61±,2.94)	Subcutaneous injections using a fanning technique (0.1–0.2 cc per trace) with a 25G x 50 mm cannula Single session	Aesthetic Safety
Bravo [7]	Sub-zygomatic area and mandibular body	Premixed product	HA+CaHA (HArmonyCa-with Lidocaine, Allergan Aesthetics)	–	2.5 mL per patient (1.25 mL for each hemiface)	Retrograde linear fanning technique, small bolus followed by retrograde injections; 21G needle 0.80 × 50 mm for perforation, 22G cannula 0.70 × 50 mm for injection Single session	Aesthetic Safety
Chang [29]	Nasolabial folds and jawline	At the same time, mixed manually before injection	HA (Belotero Balance, Anteis SA, Switzerland). CaHA (Radiesse, Merz North America)	NR	3.0 mL total (1.5 mL CaHA, 1.0 mL HA, 0.5 mL lidocaine)	Subdermal plane correction in fan shape pattern with 27-gauge cannula Single session	Aesthetic Safety
	Postauricular area	*Int:* At the same time, mixed manually before injection. *Cnt:* CaHA alone	HA (Belotero Balance, Anteis SA, Switzerland). CaHA (Radiesse, Merz North America)	NR	*Int:* 0.1 mL mixture of CaHA and HA fillers *Cnt:* 0.1 mL of only CaHA filler	Subdermal plane correction in fan shape pattern with 27-gauge cannula Single session	Mechanism
Urdiales-Galvez [27]	Preauricular region (ascending and descending mandibular branches)	Premixed product	HA+CaHA (HArmonyCa-with Lidocaine, Allergan Aesthetics)	–	1.25 mL per side	Reticular dermis, at its junction with the subcutaneous cellular tissue with a 23G cannula Single session	Aesthetic Mechanism Safety
Barone [13]	Preauricular region (ascending and descending mandibular branches)	Premixed product	*Int:* HA+CaHA (HArmonyCa-with Lidocaine, Allergan Aesthetics). *Cnt 2:* SmartXide2 DOT/RF fractional CO2 laser (DEKA®)	–	*Int:* 1.25 ml per side. *Cnt 1:* Autologous fat.	*Int:* Subcutaneous space, in contact with the undersurface of the deep dermal layer using retrograde threads with a 23G cannula Single session *Cnt 1:* Fat harvesting though liposuction with 3- Coleman aspiration cannula, centrifuged at 3000 rmp for 3 minutes, rinsed, emulsified, and filtered to obtain nano-fat. Single session *Cnt 2:* 15-Watt power, H-pulse mode, 550 μm spacing Two sessions separated by 4–6 weeks	Satisfaction Aesthetic Safety
Wortsman [30]	Majority in the mandible and the malar regions	33.3% of patients were injected with an undiluted formulation, 33.3% with diluted, and 33.3% with a premixed product	CaHA (Radiesse, Merz Pharma GmbH & Co. KGaA). HA+CaHA (HArmonyCa, Allergan Aesthetics)	–	NR	NR	Inflammation
Godin [15]	Nasolabial folds, Upper and lower lip, vermillion borders, Lipstick lines, Perioral lines, Upper and lower lip substance	CaHA followed by HAs: Concurrent injection of fillers for 6 patients and average 103 days after CaHA treatment for remaining patients	CaHA (Radiesse, BioForm Inc, Franksville, Wis). HA (Restylane, Q-Medical, Uppsala, Sweden).	**–**	*Int:* 0.9 mL (mean) of CaHA followed by 1.1 mL (mean) of HA. *Cnt*: 0.91 mL (mean) of CaHA	HA: Needle 1 inch, 30-gauge. CaHA: Needle 1.5 inch (3.8 cm), 27-gauge Single session	Satisfaction Safety
Ilaria [33]	Malar regions	HA followed by hybrid filler (HA+CaHA) (time interval was not specified)	HA (Vycross®) Hybrid filler: NM	NR	1 mL of the hybrid filler on the left and 0.25 mL on the right side	Single session	Satisfaction Safety
Yutskovskaya [28]	Periauricular area	*Group 1 (Int)*: HA immediately followed by CaHA *Group 2 (Cnt)*: HA followed by CaHA after 1 month.	HA (Belotero Volume, Merz Aesthetics, Raleigh, NC, USA). CaHA (Radiesse, Merz Aesthetics, Raleigh, NC, USA)	–	NR	Intradermal injection, periauricular area, 27 G 19 mm needle Single session	Mechanism Safety
Zerbinati [32]	Mid-face region	Premixed product	Neauvia Organic Stimulate, MatexLab SA, Lugano, CH	–	1 mL	Delivered in the deep subcutaneous layer using either a 27-gauge needle (Bolo Technique) for vertical vector corrections or a 22-gauge cannula (retrograde sliding injection technique) for homogeneous and linear infiltration in the more superficial subcutaneous layer Single session	Aesthetic Satisfaction
Somenek [12]	Perioral region	CaHA at the first visit and week 8 followed by HA at week 16	CaHA (Radiesse, Merz North America, Inc.). CPM-HA22.5 (Merz Aesthetics, Raleigh, NC)	–	*CaHA*: 3.8 ml (mean). *HA*: No information reported	*CaHA*: 1:3 dilution with 4.5 cc of sterile water using 25G 1.5-inch needle. 2 sessions *HA*: No information reported Single session	Aesthetic Safety
Felix Bravo [9]	Zygomatic-malar region, pyriform aperture, temporal region, jaw	HA immediately followed by CaHA	HA (Belotero Intense and Volume, Merz Aesthetic). CaHA (Radiesse, Merz Aesthetic)	–	HA: Up to 3 mL CaHA: 3 mL (each facial side)	*HA:* Injections were performed using a 22 G cannula through one orifice made with a 22 G needle located 5 cm down from the inferior orbital margin. Single session *CaHA:* Subcutaneously using the fan technique, a 22 G cannula after making an entry hole with a 22 G needle Single session	Aesthetic Satisfaction Safety

^1^If applicable, number of participants in intervention and control groups are reposted.

^2^Age is reported either in mean and standard deviation (SD), mean (minimum-maximum), or minimum-maximum

*CaHA* Calcium hydroxylapatite; *HA* Hyaluronic acid; *Int* Intervention; *Cnt* Control; *NR* Not reported

**Table 2:** Characteristics of non-human studies

	Author, year	Country	Study type	No. of groups	Cell/Animal type	Follow-up	Intervention (Product information)	Control	Outcomes
CR16	Zerbinati [23]	Italy	In vitro	-	Fibroblasts	-	Premixed solution, Neauvia Stimulate (MatexLab SA, Lugano, CH).	NA	Mechanism Safety
CR20	Zerbinati [34]	Italy	In vitro	-	Human keratocytes	-	Premixed solution, Neauvia Stimulate (MatexLab SA, Lugano, CH).	NA	Safety
CR7	Fan [26]	South Korea	Animal study	5	5-week-old female BALB/c nude mice	12w	Premixed solution, HA-nano-hydroxylapatite (NR) Premixed solution, HA-micro-hydroxylapatite (NR)	No treatment. CaHA, (Radiesse, Raleigh, North Carolina). HA, (Restylane, Uppsala, Sweden).	Mechanism Safety
CR10	Larkina [31]	Ukraine	Animal study	6	6–7-month-old female Wistar rats of herd breeding	60d	Premixed solution, CaHA 55.7% with HA 2% (HArmonyCA, Luminera) Premixed solution, CaHA 55.7% with HA 4%, Crystalys diluted 1:1 with Hydrial 4% (HArmonyCA, Luminera). Premixed solution, CaHA 1% with HA 2.6% (Neauvia stimulate).	Saline CaHA 55.7% diluted 1:1 with 0.9% NaCl (Radiesse, Raleigh, North Carolina) HA 4% (Hydrial 4%, Luminera)	Mechanism
CR21	Jeong [25]	South Korea	Animal study	3	5-week-old female BALB/c nude mice	12w	Premixed solution, HA-micro-hydroxylapatite (NR) Premixed solution, HA-nano-hydroxylapatite (NR)	Pure HA (Restylane, Q-MED, Galderma, Uppsala, Sweden)	Mechanism
CR8	Jeong [24]	South Korea	Animal study	2	NR	4w	Premixed solution, HA-nano-hydroxylapatite	Pure HA (NR)	Mechanism

*CaHA* Calcium hydroxylapatite; *HA* Hyaluronic acid; *NR* Not reported; *NA* Not applicable

### Quality Assessment

The potential risk of biases was evaluated in eighteen studies. One study was a case report and could not be evaluated for risk of bias. Randomized controlled trials (*n *= 2) were assessed as having an overall high risk of bias arising from the randomization process, the intended intervention, or the outcome measurement (Table S2). For the non-randomized studies (*n *= 10), all showed a serious risk of bias due to confounding factors. Many of these studies also had a serious risk of bias in the measurement of outcomes, primarily due to subjective assessment methods or unblinded evaluators (Table S5). Regarding the studies performed on animals (*n *= 4), in over 80% of the domains described in the SYRCLE’s tool, we were unable to perform a risk of bias due to lack of information in the published manuscripts (Table S3). In vitro studies (*n *= 2) received an overall medium risk of bias score (Table S4).

### Findings

#### Mechanism

Ten studies investigated potential mechanisms of combining HA and CaHA. Collagen synthesis-stimulating properties of combined therapy with HA and CaHA in human fibroblasts were shown in vitro, with the stimulating activity increasing as the concentration of the CaHA component increases [23]. Similar findings were shown in the other studies involving both animals and humans [24–29]. The included studies suggested that the combination of HA and CaHA can enhance dermal collagen formation and, consequently, the collagen fiber area through increased gene expression of the procollagen gene and heightened synthesis of collagen type 1 and type 3 proteins [24, 25, 27–29]. This effect was more pronounced when the combination was administered simultaneously rather than at different times [28] and it was also more pronounced for the combination of HA-nano-hydroxylapatite compared to HA-micro-hydroxylapatite [13, 14]. Further potential molecular mechanisms suggested by the included studies through which the combination exerts its effects include increased expression of VEGF, EGRF, and Smad2/3 mediated TGF-beta activity, which may be associated with enhanced production of both collagen and elastin fibers [26, 28]. Ultrasound studies, histological, and immuno-histological examinations showed increased dermal thickness without inflammatory or perivascular changes [7, 27, 29, 30]. Additionally, it was reported that mixtures with a higher HA concentration are associated with less inflammation, as measured by the Antioxidant–Prooxidant Index [31]. More information is provided in Table 3.

**Table 3:** Summary of results on mechanism

Author, year	Outcome/assessment method	Findings
*Human studies*		
Bravo [7]	Dermal thickness increase/high-frequency ultrasound	Dermal thickness increase (Mean ± SD, *p*-value vs. pre-treatment) Pre-treatment: 1.47 ± 0.08 mm At day 30: 1.48 ± 0.09 mm, *p *= 0.334 At day 90: 1.56 ± 0.08, *p *< 0.001 At day 120: 1.68 ± 0.08. *p *< 0.001
Chang [29]	Histologic analysis results /histological examination	Histologic analysis Combined treatment group: newly formed, irregular, thick collagen bundles in the dermis; no perivascular inflammation CaHa only group: newly formed, irregular, thick collagen bundles in the dermis; no perivascular inflammation.
Urdiales-Galvez [27]	Collagen fiber increase/strain elastography	Collagen fiber increase Pre-treatment: heterogeneous pattern with soft anechoic and hyperechoic images. Immediately after treatment: globular and poorly defined pattern; incipient 'coarse snow grain' pattern; subcutaneous cellular tissue edema. 48 hours after treatment: Globular pattern with well-defined anechoic areas; incipient 'coarse snow grain' pattern. 30 days after treatment: Total integration of HA into the tissue; 'coarse snow grain' pattern; neocollagenesis observed. 180 days after treatment: Full integration of HA; 'coarse snow grain' pattern; increase in collagen fibers evident.
Wortsman [30]	1. Inflammation/* 2. Posterior acoustic shadowing (PAS) evaluation/* 3. Vascularity evaluation/* *Ultrasound using a multifrequency probe ranging from 21 to 71 MHz (in all cases, a greyscale, a color Doppler, and a pulsed Doppler)	1. Inflammation Despite the presence of inflammatory changes in the neighboring tissues of CaHA deposits in a relevant number of undiluted and diluted CaHA deposits, which might be related to a low degree of inflammation, the mixed formulation did not present inflammatory changes in the periphery; however, this could change over time. Posterior acoustic shadowing (PAS) evaluation 2. Posterior acoustic shadowing 100% of cases with undiluted CaHA presented strong PAS, and 100% of diluted CaHA were categorized as showing mild PAS artifacts. In the mixed formulation, at 18–24 MHz, 57% showed mild PAS, and 43% showed no PAS artifact. All patients studied with 70 MHz presented CaHA as hyperechoic deposits with strong PAS. 3. Vascularity evaluation Hypervascularity was found in 43% of undiluted, 14% of diluted, and 0% of the mixed formulation.
Yutskovskaya [28]	1. Expression of collagen fibers and VEGF/Immunohistochemistry 2. Histomorphological evaluation/hematoxylin and eosin staining and Van Gieson staining	1. Expression of collagen fibers and VEGF a. Before and after comparison for separate injections [Pre-treatment (Mean) vs. At month 3 (Mean) vs at month 6 (Mean)] VEGF Expression: 0.8 vs. 4.0 vs. 4.0 Collagen Type 1 Expression: 2.0 vs. 3.5 vs. 4.5 Collagen Type 3: 2.5 vs. 4.0 vs. 4.5 b. Before and after comparison for simultaneous injections [Pre-treatment (Mean) vs. At month 3 (Mean) vs at month 6 (Mean)] VEGF Expression: 1.0 vs. 2.5 vs. 0.5 Collagen Type 1 Expression: 2.0 vs. 3.5 vs. 3.5 Collagen Type 3 Expression: 2.5 vs. 4.0 vs. 3.5 2. Expression of collagen fibers and VEGF a. Before and after comparison for separate injections [Pre-treatment (Mean) vs. At month 3 (Mean) vs at month 6 (Mean)] Elastic fiber expression: 1.0 vs. 3.0 vs. 6.0 Lymphohystiocitic infiltration: 1.0 vs. 1.5 vs. 0.5 Angiogenesis: 0.5 vs. 1.5 vs. 3.0 *r* < 0.05 b. Before and after comparison for simultaneous injections [Pre-treatment (Mean) vs. At month 3 (Mean) vs at month 6 (Mean)] Elastic fiber expression: 1.0 vs. 3.0 vs. 4.0 Lymphohystiocitic infiltration: 1.5 vs. 1.5 vs. 0.5 Angiogenesis: 0.0 vs. 3.0 vs. 1.5
*Animal studies*		
Fan [26]	1. Dermal collagen and elastic fiber formation / Real-time PCR (mRNA expression levels), 2. Western blot analysis (protein expression) 3. Immunohistochemical analysis (Verhoeff–Van Gieson staining)	1. Real-time PCR, mRNA expression levels HA-micro-hydroxylapatite group: EGFR, Smad2, procollagen, elastin, and fibrillin mRNA expression levels exhibited similar patterns. The procollagen levels were significantly higher in the HA-micro-hydroxylapatite group than in the Radiesse group (*p* < 0.005). The Smad2 and fibrillin levels of the HA-micro-hydroxylapatite group were significantly higher than those of the Radiesse group (*p* < 0.05), whereas the Smad3 levels were significantly lower (*p* < 0.05). No significant differences in the expression of any gene were observed between groups at week 12, at which time the gene expression levels decreased. HA-nano-hydroxylapatite group: At week 4, the EGFR, Smad2, procollagen, elastin, and fibrillin mRNA expression levels had similar patterns, whereas no significant differences were observed for Smad3. The EGFR and elastin levels of the HA-nano-hydroxylapatite group were significantly higher than those of the Restylane group (*p* < 0.005). No significant differences in the expression of any gene were observed between groups at week 12, at which time the gene expression levels decreased. 2. Western blot analysis—Protein expression HA-micro-Hap group showed higher protein expression for TGF-β, EGFR, p-MAPK, Smad2/3, and collagen type 1 expression, whereas the Radiesse group did not. HA-nano-Hap group: Western blotting at 12 weeks after filler injections showed that the Restylane and HA-nano-hydroxylapatite groups had similar levels of TGF-*β*, EGFR, p-MAPK, Smad2/3, and collagen type 1 although the EGFR, p-MAPK, and collagen type 1 levels of the HA-nano-hydroxylapatite group increased significantly compared to those in controls (*P* < 0.005). Smad7 expression was negatively correlated with Smad2/3 protein expression in both groups. 3. Verhoeff–Van Gieson staining—immunohistochemical analysis Both HA-micro-Hap and HA-nano-hydroxylapatite showed increased elastic fiber synthesis compared to Radiesse (*p *< 0.05)
Larkina, 2021 [31]	1. Malondialdehyde (MDA) Content/Reaction with 2-thiobarbituric acid 2. Acid Phosphatase Activity/Bessey et al. method on the hydrolysis of n-nitrophenyl phosphate 3. Neutrophil Elastase Activity/Visser and Blout method by the hydrolysis of N-t-BOC-L-alanin-p-nitrophenyl ester 4. Antioxidant-Prooxidant Index (API) / Ratio of catalase activity to MDA content	1. Malondialdehyde (MDA) Content, mmol/kg (Mean±SD), *p*-value vs. Control) CaHA 55.7% with HA 2%: 3.01 ± 0.12, *p *< 0.002 CaHA 55.7% with HA 4%: 2.09 ± 0.15, *p *> 0.3 CaHA 1% with HA 2.6%: 3.34 ± 0.21, *p* < 0.001 2. Acid Phosphatase Activity, k-cat/kg (Mean±SD, p-value vs. control) CaHA 55.7% with HA 2%: 15.34 ± 1.0, 0.05 CaHA 55.7% with HA 4%: 11.09 ± 0.86, *p *> 0.25 CaHA 1% with HA 2.6%: 13.6 ± 0.78, *p *> 0.5 3. Neutrophil Elastase Activity, mk-cat/kg (Mean ± SD, *p*-value vs. control) CaHA 55.7% with HA 2%: 16.18 ± 1.13, *p *< 0.01 CaHA 55.7% with HA 4%: 12.16 ± 0.97, *p* > 0.8 CaHA 1% with HA 2.6%: 15.41 ± 1.25, *p* <0.02 4. Antioxidant-Prooxidant Index (API), μcat/kg (Mean±SD, *p*-value vs. control) CaHA 55.7% with HA 2%: 2.26 ± 0.10, *p* > 0.4 CaHA 55.7% with HA 4%: 2.83 ± 0.14, *p* < 0.05 CaHA 1% with HA 2.6%: 2.56 ± 0.13, *p* > 0.4
Jeong, 2016 [25]	1. Dermal thickness/H&E staining 2. Procollagen gene expression/Real-time PCR (relative quantity)	1. Dermal thickness (Mean) Pure HA group: 180 μm HA-micro-hydroxylapatite: 200 μm HA-nano-hydroxylapatite: 210 μm 2. Procollagen gene expression (Mean) Pure HA group: 1 HA-micro-hydroxylapatite: 1.6 HA-nano-hydroxylapatite: 2.7
Jeong [24]	1. Collagen fiber area/Masson’s trichrome staining 2. Elastic fiber area/Verhoeff-Van Gieson staining	1. Collagen fiber area Significant increase in collagen area after HA-nano-hydroxylapatite injection compared with both UVB- and pure HA-treated groups (*p *< 0.05) 2. Elastic fiber area HA-nano-hydroxylapatite group showed larger proportion than pure HA group (*p *< 0.01)
*In vitro studies*		
Zerbinati [23]	Collagen synthesis in human fibroblasts/Colorimetric assay	Collagen synthesis in human fibroblasts At baseline (Mean): 60 μg/ml 24 h post-incubation (Mean): HA-CaHA (1.25 mg/ml): ~ 80 μg/ml HA-CaHA (2.5 mg/ml): ~ 120 μg/ml

*HA* Hyaluronic acid; *CaHA* Calcium hydroxylapatite

#### Aesthetic Outcomes

Eight studies reported aesthetic-related outcomes [7–9, 12, 27, 29, 32]. These studies demonstrated improvement in both investigator and subject-reported aesthetic outcomes, with no studies reporting worse aesthetic results following the intervention. One study reported 4.6/5 on the Visual Analog Scale [13], and two studies reported 100% of investigators observing at least some improvement on the Global Aesthetic Improvement Scale [9, 32]. Aesthetic improvements were reported in various facial areas, such as jawline [7–9, 27, 29], zygomatic and malar region [7, 9, 32], perioral region [12], and nasolabial folds [29]. Only four of these eight studies had a follow-up period of more than six months [8, 27, 29, 32]. These studies indicated a trend of decreased aesthetic outcomes and improvements after this time point, but the results were still rated as satisfactory. Also, the combination treatment was shown to improve facial laxity and provide facial volume and a lifting effect [9, 27, 32]. Results are summarized in Table 4.

**Table 4:** Summary results on aesthetic effectiveness

Author, year	Treated region	Outcome/assessment method	Findings
Barone [13]	Preauricular region (ascending and descending mandibular branches)	1. Aesthetic core (Investigator)/VAS Scale 2. FACE-Q Questionnaire (Subjects)	1. Aesthetic score (Investigator) *Overall aesthetic score (M*ean score) *Autologous fat group*: 4.7 *HA-CaHA*: 4.6 CO_2_* laser resurfacing*: 3.9 *Subject satisfied with the face after treatment—Autologous fat injection (n, %)* Symmetry: 16 participants (80%) Balanced face: 16 participants (80%) Face proportion: 17 participants (85%) Face at the end of your day: 17 participants (85%) Freshness of the face: 18 participants (90%) Rested face: 17 participants (85%) Profile: 17 participants (85%) Looking in photos: 19 participants (95%) Looking when wake-up: 16 participants (80%) Looking under bright lights: 18 participants (90%) *Subject satisfied with the face after treatment—HA-CaHA (n, %)* Symmetry: 18 participants (82%) Balanced face: 17 participants (77%) Face proportion: 18 participants (82%) Face at the end of your day: 17 participants (77%) Freshness of the face: 18 participants (82%) Rested face: 16 participants (69%) Profile: 19 participants (86%) Looking in photos: 20 participants (91%) Looking when wake-up: 18 participants (82%) Looking under bright lights: 19 participants (86%) *Subject satisfied with the face after treatment—Fractional *CO_2_* laser resurfacing (n, %)* Symmetry: 11 participants (48%) Balanced face: 12 participants (52%) Face proportion: 18 participants (78%) Face at the end of your day: 15 participants (65%) Freshness of the face: 13 participants (56%) Rested face: 16 participants (69%) Profile: 11 participants (48%) Looking in photos: 14 participants (61%) Looking when wake-up: 12 participants (52%) Looking under bright lights: 15 participants (65%) 2. FACE-Q (Subjects) *Autologous fat injection (Mean score)* Global cosmetic outcome: 4.7 Scarring: 5 Profile view: 4.8 Frontal view: 4.9 Basal view: 4.8 Feminine/masculine shape: 5 *HA-CaHA (Mean score)* Global cosmetic outcome: 4.6 Scarring: 5 Profile view: 4.8 Frontal view: 4.9 Basal view: 4.8 Feminine/masculine shape: 5 *Fractional *CO_2_* laser resurfacing (Mean score)* Global cosmetic outcome: 3.9 Scarring: 4.9 Profile view: 3.9 Frontal view: 3.8 Basal view: 3.8 Feminine/masculine shape: 5
Bravo [9]	Zygomatic-malar region, pyriform aperture, temporal region, jaw	1. Improvement in facial skin laxity (Investigator)/Global Aesthetic Improvement Scale 2. Improvement in facial skin laxity (Subject)/Global Aesthetic Improvement Scale 3. Dermal thickness/High-frequency ultrasonography 4. Other aesthetic parameters (Participants and Investigators)	1. Improvement in facial skin laxity (Investigator) The consensual clinical evaluation by physicians comparing the pre-treatment and day 90 after treatment images resulted in one (7%) improved, 9 (60%) very improved, and five (33%) exceptionally improved patients. 120 days after treatment, PGAIS resulted in six (40%) very improved patients and nine (60%) exceptionally improved patients. 2. Improvement in facial skin laxity (Subject) 100% Exceptionally Improved 3. Dermal thickness At day 30: 0.6% increase (0.2–1.0%) At day 90: 5.7% increase (4.2–7.3%), At day 120: 11.1% increase (8.8–13.4%) The superior performance of the treatment compared with the control side (no treatment) was detected from day 90 (*P* < 0.01). 4. Other aesthetic parameters Facial laxity All the participants evidenced improvement in facial laxity based on a combination of volume replacement in the malar area, an increase in dermal thickness, and an improved definition of the mandibular angle and a sharper contour. Facial volume The evaluation of the facial volume changes through the comparison of the images from baseline and day 120 revealed an increase in malar mass. Lifting effect: Analysis of the vectors provided by the comparison of baseline and day 120 images revealed a significant lifting effect on the face.
Bravo [7]	Sub-zygomatic area and mandibular body	1. Overall Aesthetic Improvement (Investigator)/Global Aesthetic Improvement Scale (1-worse to 5 -very much improved) 2. Improvement Scale (Subjects) (1-worse to 5 -very much improved)	1. Overall Aesthetic Improvement (Investigators) At Day 30: 15 patients were rated as Very Much Improved, 0 were rated as Much Improved, 0 were rated as Improved, 0 were rated as Mildly Improved, and 0 were rated as Worse. At Day 60: 15 patients were rated as Very Much Improved, 0 were rated as Much Improved, 0 were rated as Improved, 0 were rated as Mildly Improved, and 0 were rated as Worse. At Day 90: 14 patients were rated as Very Much Improved, 1 was rated as Much Improved, 0 were rated as Improved, 0 were rated as Mildly Improved, and 0 were rated as Worse. At Day 120: 14 patients were rated as Very Much Improved, 1 was rated as Much Improved, 0 were rated as Improved, 0 were rated as Mildly Improved, and 0 were rated as Worse. 2. Overall Aesthetic Improvement Subjects (%) At Day 120: 20% of patients showed Exceptional Improvement, 20% were Very Improved, and 60% were Improved.
Chang [29]	Nasolabial folds and jawline	1. Jawline contour improvement (Investigator)/Visual Analog Scale (1-very poor to 10—excellent) 2. Nasolabial fold improvement (Investigator)/Visual Analog Scale (VAS, 1-very poor to 10—excellent) 3. Jawline contour improvement (Investigator)/5-point global satisfaction scale (1—very dissatisfied to 5—very satisfied) 4. Nasolabial fold improvement (Investigator)/(1—very dissatisfied to 5—very satisfied)	1. Jawline contour improvement (Mean score) At month 1: 7.4 At month 2: 7.4 At month 3: 6.7 At month 9: 6.4 2. Nasolabial fold improvement (Mean score) At month 1: 7.0 At month 2: 6.9 At month 3: 6.0 At month 9: 5.8 3. Jawline contour (Mean score) At month 1: 4.7 At month 2: 4.2 At month 3: 3.6 At month 9: 3.2 4. Nasolabial fold improvement (Mean score) At month 1: 4.8 At month 2: 4.4 At month 3: 3.8 At month 9: 3.4
Fakih [8]	Mid- and lower face	Jawline Aesthetics Score (Investigator)/Merz Aesthetics Scale for jawline	Jawline Aesthetics Score (Investigators), (Mean ± SD, *p*-value vs. baseline) At baseline: 2.12 ± 0.81 At month 3: 0.68 ± 0.69, *p *< 0.001 At month 12: 1.27 ± 0.74, *p *< 0.001
Somenek [12]	Perioral region	1. Improvement in perioral rhytids (Investigator)/Merz perioral/lip wrinkle grading scale (1—no wrinkles to 5—very severe wrinkles) 2. Improvement in perioral rhytids (Subjects)/NR 3. Aesthetic improvement (Investigator)/Global Aesthetic Improvement Scale (GAIS) 4. Aesthetic improvement (Subject)/Global Aesthetic Improvement Scale (GAIS)	1. Improvement in perioral rhytids (Investigator) Investigator ratings based on the 5-point Merz perioral/lip wrinkles grading scale showed at least 1 grade improvement in 83% of the patients. Mean difference (from baseline): 0.82 (CI 95% 0.23-1.32, *p *= 0.0156) 2. Improvement in perioral rhytids (Subjects) A majority (75%) rated their lip wrinkle as improved between Visit 1 (baseline) and 4 (week 20). 3. Aesthetic improvement (Investigator) At week 20: 9% not improved, 36% improved, 36% much improved, 18% very much improved 4. Aesthetic improvement (Subject) At week 20: 18% not improved, 55% improved, 18% much, 9% very much improved
Urdiales-Galvez [27]	Preauricular region (ascending and descending mandibular branches)	1. Volumetric Changes (Investigator)/3D photographs 2. Lifting effect (Investigator)/Facial Tension Vectors—3D photographs	1. Volumetric changes (Investigator, cc (Median (Inter quartile range), p-value vs. baseline) *Right Side:* At 48 hours: 1.7 (1.3–2.0), *p *< 0.0001 At day 30: 1.7 (1.4–1.9), *p* < 0.0001 At day 90: 1.8 (1.3–2.1), *p *< 0.0001 At day 180: 2.1 (1.9–2.3), *p *< 0.0001 *Left side:* At 48 hours: 1.7 (1.3–1.9), *p* < 0.0001 At day 30: 1.4 (0.9–1.9), *p *< 0.0001 At day 90: 1.9 (1.6–2.1), *p *< 0.0001 At day 180: 2.1 (1.8–2.2), *p *< 0.0001 2. Lifting effect (Investigator) Median (Inter quartile range), *p*-value vs. baseline *Right Side* Day 60,1.9 (1.3–2.4), *p *< 0.0001 Day 90,2.2 (1.7–2.5), *p *< 0.0001 Day 180,2.2 (1.6–2.2), *p *< 0.0001 *Left Side* At day 60: 2.1 (1.4–2.5), *p *< 0.0001 At day 90: 2.1 (1.8–2.5), *p *< 0.0001 At day 180: 2.0 (1.7–2.2), *p *< 0.0001
Zerbinati [32]	Mid-face region	1. Mid-face aesthetic improvement (Investigator)/Mid-face volume deficit scale 2. Aesthetic improvement (Investigator)/Global Aesthetic Improvement Scale	1. Mid—face aesthetic improvement score (Investigator) (Mean±SD, *p*-value vs. Baseline) At baseline: 3.2 ± 0.443 Immediately post-treatment: 1.77 ± 673 At month 1: 1.33 ± 0.542 At month 3: 1.40 ± 0.588 At month 6: 2.62 ± 0.555, *p *< 0.05 2. Global aesthetic improvement (Investigator) At week 4: 28 patients (46%)—very much improved, 21 patients (35%)—improved, 11 patients (18.3%)—improved. No patient was considered “Worse” following treatment. At week 12: 21 patients (35%)—very much improved, 22 patients (36.7%)—improved, 16 patients (26.7%)—improved. One patient (1.7%) was judged to be “No change”. No patient was considered “Worse” at this time point. At week 24: 0 patients were still considered “Very much improved”, 9 patients (15%)—much improved, 33 patients (55%) were judged to be “No change” and no patients were considered ‘Worse’. Time point, score (Mean) Immediately post-treatment: 2.27 At month 1: 1.72 At month 3: 1.95 At month 6: 3.40

*HA* Hyaluronic acid; *CaHA* Calcium hydroxylapatite

#### Satisfaction

Among four studies [9, 15, 32, 33] reporting on satisfaction, all unanimously reported high satisfaction scores. One study reported 100% of patients as being at least satisfied with the treatment, one study [15] reported an overall satisfaction of 8.1/10 and one study [9] reported a satisfaction score of 8/10. Nasolabial folds and perioral region were the treated areas with the more satisfactory results with satisfaction scores 8.2/10 on a 10-point scale. However, the chin area was associated with the least satisfactory outcomes in one study with scores 5.0/10 reported [24]. This study noted that satisfaction with the treatment decreased with longer follow-up periods. A similar trend was observed in another study [32], where satisfaction decreased after six months of follow-up, suggesting a time-dependent effect. Table 5 summarizes these findings.

**Table 5:** Summary of results: satisfaction

Author, year	Treated region	Outcome/assessment method	Findings
Godin [15]	Nasolabial folds, Upper and lower lip, vermillion borders, Lipstick lines, Perioral lines, Upper and lower lip substance	Site-specific satisfaction (Subject)/Satisfaction survey (10-point scale)	Site-specific satisfaction (Subject) (Mean score) Nasolabial fold: 8.2 Upper vermillion border: 7.7 Upper lip substance: 7.0 Lower vermillion border: 6.5 Lower lip substance: 6.0 Lipstick lines: 7.2 Perioral region: 7.2 Chin: 5.0
		General satisfaction (Subject)/Satisfaction survey (10-point scale)	General satisfaction (Subject) (Mean score) Overall satisfaction: 8.1 Immediate satisfaction: 8.4 Long-term satisfaction: 6.5 Of the 15 patients who responded to the survey, 13 (86%), 13 (86%), and 10 (69%) rated their overall, immediate, and long-term satisfaction with Radiesse and Restylane combination as either excellent or good, respectively; 12 (79%) of the 15 respondents would recommend this combination to a friend.
Ilaria [33]	Various facial regions	Satisfaction/Comment	Satisfaction The outcome was highly satisfactory.
Bravo [9]	Zygomatic arch, zygomatic eminence, the mid malar region, and the deep malar fat compartment	Satisfaction (Subject)/5-point scale	Satisfaction (Subject) All reported scores of 5 (highly satisfied) at day 120.
Zerbinati [32]	Mid-face region	Satisfaction (Subject)/Visual Analogue Scale	Satisfaction (Subject) (Mean score) Immediately after treatment: 8.3 At months 1: 8.5 At month 3: 8.3 At month 6: 8
		Satisfaction (Investigator)/Visual Analogue Scale	Satisfaction (Investigator) (Mean score) Immediately after treatment: 8.6 At months 1: 8.8 At month 3: 8.8 At month 6: 8.5

*HA* Hyaluronic acid; *CaHA* Calcium hydroxylapatite

#### Safety Profile

Eight studies reported safety following treatment with HA and CaHA [7–9, 11–15, 27–29, 33]. Studies showed mild and manageable adverse events, including mild to moderate treatment-emergent events such as pain, edema, implant site nodules, inflammation, hypersensitivity, skin induration, and ecchymosis. Most adverse events resolved quickly, either spontaneously or with minimal treatment such as topical creams. No severe complications such as infections, vascular complications, or granulomas were observed [7–9, 11–15, 27–29, 33]. Overall, the combined intervention was generally well-tolerated with a low incidence of severe adverse events, and animal and in vitro studies showed no significant adverse effects, such as organ damage or cytotoxicity [23, 26, 34]. Safety profile following combined hybrid treatments is summarized in Table 6.

**Table 6:** Summary of results on safety and adverse effects

Author, year	Findings
*Human studies*	
Kadouch [11]	The only events reported were 2 cases of slight overcorrection in a small area at 1-3 months. Both involved high volumes of CaHA and CPM-HA V (4.5 and 8 cc, respectively, in one patient, and 6 and 7 cc), but resolved with 60 U hyaluronidase (Hyalase) and were not visible at subsequent follow-up visits. No other AEs were reported.
Braz [14]	Mild TEAEs were reported by 14 participants (3.5%) and moderate by 5 (1.2%). Documented TEAEs included edema, implant site nodule, inflammation, hypersensitivity, and skin induration. There were no reports of vascular complications or the Tyndall effect. The duration of TEAEs in the full analysis set ranged from less than 1 month to 12 months. All TEAEs but two were documented as resolved by study end, though investigators confirmed resolution of these remaining TEAEs after database lock. No participants in the full analysis set experienced TEAEs of grades 3, 4, or 5 severities, and no participant had a documented TEAEs. Safety outcomes were similar among the long-term safety analysis, with 15 participants (6.2%) having a total of 16 documented TEAEs; the majority were mild in severity (n = 10 participants; 4.1%). Six (2.5%) participants reported edema that was mild (n = 4, 1.6%) or moderate (n = 2, 0.8%) in severity. Five (2.1%) participants reported implant site nodules that were mild (n = 4, 1.6%) or moderate (n = 1, 0.4%) in severity. One (0.4%) participant reported inflammation that was moderate in severity, three (1.2%) participants reported skin induration that was mild in severity, and one (0.4%) participant experienced hypersensitivity that was moderate in severity.
Fakih-Gomez [8]	No adverse events other than injection site reactions were reported for any of the subjects at any given time.
Bravo [7]	No infections, ischemia, or other relevant adverse effects were observed, but mild pain, ecchymosis, and local edema occurred after the injections. In one patient, there was an adverse event considered mild, in which a whitish papule appeared due to the accumulation of the product in the cannula inlet. Localized and self-limited side effects such as mild edema, pain, and ecchymosis were seldom reported. No vascular occlusive events or infections following the procedure were noted. Also, no clinical or sonographic signs of granuloma formation were detected in this assessment. Only one patient had an adverse event, developing a small papule in the lower face.
Chang [29]	There were no adverse events such as inflammation, skin problems, nodular formation, or granuloma during the follow-up period.
Urdiales-Galvez [27]	There were neither unexpected nor serious treatment-related adverse events. Most patients experienced a mild redness and inflammation that resolved within the first 48 h without treatment. Three (20%) patients had an ecchymosis, which was successfully solved with topical treatment (Arnica+Vit K cream). Four (26.7%) patients reported discomfort immediately after treatment, which resolved satisfactorily without treatment. All the reported adverse events were mild in severity, and all were fully recovered with topical treatment.
Barone [13]	No major adverse events were reported.
Godin [15]	Most patients experienced mild to no pain during treatment. Two (13%) patients reported downtime longer than 24 hours. Minor sequelae resolved quickly in three out of four subjects experiencing them. Seven (47%) patients experienced swelling.
Ilaria [33]	No adverse events were reported.
Yutskovskaya [28]	No adverse events were reported.
Somenek [12]	The only adverse event that was documented was bruising postinjection, and it was categorized as mild in all instances.
Bravo [9]	No infections, nodules, ischemia, or other relevant adverse effects were noted, but mild pain, ecchymosis, and local edema occurred after the injections.
*Animal studies*	
Fan [26]	No organ damage, structural malformation, necrosis, abnormal inflammatory cell infiltration, or abnormal increase in macrophages for both HA-micro-hydroxylapatite and HA-nano-hydroxylapatite.
*In vitro studies*	
Zerbinati [23]	No cytotoxicity observed.
Zerbinati [34]	No cytotoxicity, morphological or structural changes observed.

*CPM-HA V* Cohesive polydensified matrix (CPM®, Belotero® Volume); *EAEs* Treatment-emergent adverse events; *HA* Hyaluronic acid; *CaHA* Calcium hydroxylapatite

## Discussion

To our knowledge, this is the first comprehensive systematic review aimed to evaluate the combined and hybrid treatments of HA and CaHA in terms of their mechanism of action, aesthetic effectiveness, satisfaction, and safety. In vitro and in vivo studies suggested that the combined and hybrid treatments can stimulate collagen synthesis, consistent with previously described molecular mechanisms. Overall, the studies showed improvement in aesthetic effectiveness, satisfaction following treatment and a safe profile with minor adverse events.

In vitro and in vivo studies suggested that the combination of HA and CaHA stimulate collagen synthesis with less inflammation, which is in line with the proposed molecular mechanisms involving increased expression of VEGF, EGFR, and TGF-beta activity [4, 6]. These growth factors have been shown to improve aging parameters in skin and induce tissue healing and rejuvenation. [35–37] Studies including only CaHA have shown increased expression of VEGF and EGFR, but not of TGF-beta activity [6]. However, the latter has been shown to be activated by HA [38]. These findings may suggest that the combination treatment may have more synergistic effects and thus not only by providing immediate volumizing effects but also promoting long-term skin quality improvements through collagen synthesis.

Furthermore, aesthetic improvements and high satisfaction were observed in studies, aligning with previous studies on the separate use of CaHA and HA [5, 39–42]. Among included studies, aesthetic improvements were sustained for up to six months, after which a slight decline began to appear [8, 27, 29, 32]. This trend aligns with the satisfaction outcomes and might suggest a temporary effect of the combination treatment, and whether there is a need for repeat treatments or maintenance therapy remains to be explored in future studies. Also, the decrease in satisfaction over time underscores the importance of managing patient expectations regarding the duration of treatment effects.

The studies highlighted that the combined and hybrid use of HA and CaHA is generally well-tolerated with a low incidence of severe adverse events. The mild to moderate adverse events reported, such as pain, edema, nodules, and inflammation, were mostly manageable and resolved spontaneously or with minimal treatment. These results are consistent with previous studies on dermal fillers, suggesting that the combination therapy does not introduce additional safety concerns compared to monotherapies [43, 44]. The lack of long-term safety data and the predominance of studies with short follow-up periods and high risk of bias necessitate cautious interpretation of these findings. Future research should focus on long-term safety outcomes to fully establish the safety profile of this combination therapy.

### Limitations

The findings provide valuable insights into the efficacy and safety of this combination treatment, although several limitations should be acknowledged.

The heterogeneity in study designs, absence of control groups, variations in CaHA concentration, and timing of administration, particularly regarding the mechanistic aspects and aesthetic effectiveness, complicates the synthesis of results. Furthermore, the reliance on subjective aesthetic assessment methods and the lack of standardized outcome measures present challenges in objectively evaluating aesthetic effectiveness. The standardized use of objective imaging and measurement techniques, such as three-dimensional imaging and digital morphometry, could provide more precise and reliable evaluations of aesthetic outcomes. Additionally, the predominance of studies with short follow-up periods necessitates cautious interpretation of findings, particularly regarding safety outcomes. Comprehensive long-term studies are essential to accurately assess the safety profile and ensure the sustained efficacy of these treatments. We also suggest the evaluation of the combined hybrid treatments against other commonly used dermal fillers and rejuvenation techniques and exploring the potential need for repeated treatments or maintenance therapy over time to achieve optimal efficacy. It also may be of potential interest to explore the combination treatment in areas of the body other than the face. Additionally, investigating the dose–response relationship and identifying optimal concentrations of CaHA and HA to maximize therapeutic and cosmetic benefits without increasing the risk of adverse events would be valuable. Our review also revealed a limited number of studies investigating the mechanisms underlying the synergistic effects of combination therapy. This highlights significant evidence gaps in this area and underscores the need for future mechanistic studies to explore these interactions in greater depth**.**

## Conclusion

The combinations and hybrid treatments of HA and CaHA appear to be a promising, safe, and efficacious treatment for facial rejuvenation, offering both immediate volumizing effects and longer-term benefits through collagen synthesis. While the combinations and hybrid treatments are in generally well-tolerated and associated with high satisfaction rates, its effects may diminish over time. The findings of this review provide a foundation for future research to optimize treatment protocols and establish robust evidence for the long-term safety and efficacy of this combination therapy in aesthetic medicine.

## Supplementary Information

Below is the link to the electronic supplementary material.

Supplementary file1 (DOCX 42 kb)
