# Supplementary material for: Combined and Hybrid Treatments of Hyaluronic Acid (HA) and Calcium Hydroxylapatite (CaHA): A Systematic Review of Mechanisms of Action, Aesthetic Effectiveness, Satisfaction, and Safety Profile
Source: Aesthetic Plast Surg. 2025 Jun 6;49(19):5292–313. doi: 10.1007/s00266-025-04904-x (PMC12594701; doi:10.1007/s00266-025-04904-x)
Supplement: Supplementary file 1 — Supplementary file1 (DOCX 42 kb) [file 266_2025_4904_MOESM1_ESM.docx]

**Supplementary materials: Combined treatment of hyaluronic acid and calcium hydroxylapatite: Systematic reviews of aesthetic effectiveness, mechanism of action, and safety profile**

*Table S1. Designed search strategies.*

| **Database** | **Search strategy** |
| --- | --- |
| **Medline ALL (Ovid)** | ((belotero* OR ((cohesi*) ADJ3 (polydens* OR poly-dens*)) OR estheli* OR forteli* OR modelis* OR mesolis* OR (((combi* OR mix* OR hybrid* OR cosmetic* OR injectable*) ADJ3 (filler* OR filling*)) AND (cosmetic* OR dermal* OR hand OR hands OR lip OR lips OR stretch-mark* OR finger OR fingers OR beautificat* OR skin OR derm* OR epiderm* OR dermal* OR wrinkle* OR face OR facial OR hand OR hands OR cheek* OR buttock* OR jaw* OR neck OR elbow* OR foot OR feet OR decolle* OR arm OR arms OR leg OR legs OR forehead* OR ear OR ear OR fold OR folds OR line OR lines OR chin OR eyelid* OR lip OR lips OR abdom* OR nonfacial* OR tigh OR tighs OR chest OR chests OR orbit* OR periorbit* OR submentum OR submental OR brow OR brows OR rejuvenat* OR rhinoplast* OR nose OR esthetic* OR aesthetic* OR soft-tissue* OR augmentat* OR non-human* OR invitro* OR in-vitro* OR invivo* OR in-vivo* OR animal* OR hamster* OR mouse OR mice OR rat OR rats OR rabbit* OR monkey*))).ab,ti,kf.) OR (("Hyaluronic Acid"/ OR (hyaluron*-acid* OR hyaluronate*).ab,ti,kf.) AND ("Durapatite"/ OR (radiesse* OR CaHA OR calcium-hydroxyapatit* OR calcium-hydroxylapatit*).ti,ab,kf.)) **NOT** (news OR congres* OR abstract* OR book* OR chapter* OR dissertation abstract*).pt. |
| **Embase** | ('belotero'/exp OR (belotero* OR ((cohesi*) NEAR/3 (polydens* OR poly-dens*)) OR estheli* OR forteli* OR modelis* OR mesolis* OR (((combi* OR mix* OR hybrid* OR cosmetic* OR injectable*) NEAR/3 (filler* OR filling*)) AND (cosmetic* OR dermal* OR hand OR hands OR lip OR lips OR stretch-mark* OR finger OR fingers OR beautificat* OR skin OR derm* OR epiderm* OR dermal* OR wrinkle* OR face OR facial OR hand OR hands OR cheek* OR buttock* OR jaw* OR neck OR elbow* OR foot OR feet OR decolle* OR arm OR arms OR leg OR legs OR forehead* OR ear OR ear OR fold OR folds OR line OR lines OR chin OR eyelid* OR lip OR lips OR abdom* OR nonfacial* OR tigh OR tighs OR chest OR chests OR orbit* OR periorbit* OR submentum OR submental OR brow OR brows OR rejuvenat* OR rhinoplast* OR nose OR esthetic* OR aesthetic* OR soft-tissue* OR augmentat* OR non-human* OR invitro* OR in-vitro* OR invivo* OR in-vivo* OR animal* OR hamster* OR mouse OR mice OR rat OR rats OR rabbit* OR monkey*))):ab,ti,kw) OR (('hyaluronic acid'/exp OR (hyaluron*-acid* OR hyaluronate*):ab,ti,kw) AND ('hydroxyapatite'/exp/mj OR (radiesse* OR CaHA OR calcium-hydroxyapatit* OR calcium-hydroxylapatit*):ti,ab,kw)) **NOT** ([Conference Abstract]/lim) |
| **Web of Science Core Collection** | TS=(belotero* OR ((cohesi*) NEAR/2 (polydens* OR poly-dens*)) OR estheli* OR forteli* OR modelis OR mesolis* OR (((combi* OR mix* OR hybrid* OR cosmetic* OR injectable*) NEAR/2 (filler* OR filling*)) AND (cosmetic* OR dermal* OR hand OR hands OR lip OR lips OR stretch-mark* OR finger OR fingers OR beautificat* OR skin OR derm* OR epiderm* OR dermal* OR wrinkle* OR face OR facial OR hand OR hands OR cheek* OR buttock* OR jaw* OR neck OR elbow* OR foot OR feet OR decolle* OR arm OR arms OR leg OR legs OR forehead* OR ear OR ear OR fold OR folds OR line OR lines OR chin OR eyelid* OR lip OR lips OR abdom* OR nonfacial* OR tigh OR tighs OR chest OR chests OR orbit* OR periorbit* OR submentum OR submental OR brow OR brows OR rejuvenat* OR rhinoplast* OR nose OR esthetic* OR aesthetic* OR soft-tissue* OR augmentat* OR non-human* OR invitro* OR in-vitro* OR invivo* OR in-vivo* OR animal* OR hamster* OR mouse OR mice OR rat OR rats OR rabbit* OR monkey*))) OR TS=((hyaluron*-acid* OR hyaluronate*) AND (radiesse* OR CaHA OR calcium-hydroxyapatit* OR calcium-hydroxylapatit*)) **NOT** DT=(Meeting Abstract OR Meeting Summary) |
| **Cochrane CENTRAL** | ((belotero* OR ((cohesi*) NEAR/3 (polydens* OR poly NEXT/1 dens*)) OR estheli* OR forteli* OR modelis* OR mesolis* OR ((combi* OR mix* OR hybrid* OR cosmetic* OR injectable*) NEAR/3 (filler* OR filling*))):ab,ti,kw) OR (((hyaluron* NEXT/1 acid* OR hyaluronate*):ab,ti,kw) AND ((radiesse* OR CaHA OR calcium NEXT/1 hydroxyapatit* OR calcium NEXT/1 hydroxylapatit*):ti,ab,kw)) |

Table S2. Risk of bias according to Cochrane Collaboration’s Tool Risk of Bias 2 (ROB2) for randomized trials.

| **Author, Year** | **Bias arising from** | | | | | **Overall score** |
| --- | --- | --- | --- | --- | --- | --- |
|  | **Randomization process** | **Intended interventions** | **Missing data** | **Outcome measurement** | **Selection of the reported result** |  |
| **Barone, 2024 (13)** | Some concerns | Some concerns | Lower risk of bias | Lower risk of bias | Lower risk of bias | Higher risk of bias |
| **Yutskovskaya, 2024 (46)** | Some concerns | Some concerns | Lower risk of bias | Some concerns | Lower risk of bias | Higher risk of bias |

| **TYPE OF BIAS** | **Selection bias** | | **Selection bias** | **Performance bias** | | **Detection bias** | | **Attrition bias** | **Reporting bias** | **Other** |
| --- | --- | --- | --- | --- | --- | --- | --- | --- | --- | --- |
| **DOMAIN** | **Sequence generation** | **Baseline characteristics** | **Allocation concealment** | **Random housing** | **Blinding** | **Random outcome assessment** | **Blinding** | **Incomplete outcome data** | **Selective outcome reporting** | **Other sources of bias** |
| Fan, 2019 (26) | Unclear | Yes | Unclear | Unclear | Unclear | Unclear | Unclear | Yes | Unclear | Unclear |
| Larkina, 2021 (31) | Unclear | Unclear | Unclear | Unclear | Unclear | Unclear | Unclear | Yes | Unclear | Unclear |
| Jeong, 2016 (25) | Unclear | Yes | Unclear | Unclear | Unclear | Unclear | Unclear | Yes | Unclear | Unclear |
| Jeong, 2017 (24) | Unclear | Unclear | Yes | Unclear | Yes | Yes | Unclear | Yes | Unclear | Unclear |

Table S3. Risk of bias according to SYRCLE’s risk of bias tool for animal studies.

.

*Table S4. Risk of bias according to QUIN tool for assessing in vitro studies.*

| **Author, year** | **Risk of bias** |
| --- | --- |
| Zerbinati, 2017 (23) | Medium Risk of Bias |
| Zerbinati, 2018 (35) | Medium Risk of Bias |
|  |  |

Table S5. Risk of bias according to ROBINS-I: a tool for assessing risk of bias in non-randomised studies of interventions.

| **Author, year** | **Pre-Intervention** | | **At Intervention** | **Post-Intervention** | | | |
| --- | --- | --- | --- | --- | --- | --- | --- |
|  | **Bias due to/in** | | **Bias in** | **Bias due to/in** | | | |
|  | **Confounding** | **Selection of participants** | **Classification of intervention** | **Deviations from intended interventions** | **Missing data** | **Measurement of outcomes** | **Selection of the reported result** |
| Fakih-Gomez, 2021 (8) | Serious | Low | **Serious** | Low | Low | Serious | Low |
| Bravo, 2023 (7) | Serious | Low | Low | Low | Low | Low | Low |
| Chang, 2020 (29) | Serious | Low | Low | Low | Low | Serious | Low |
| Urdiales-Galvez, 2023 (27) | Serious | Low | Low | Low | Low | Low | Low |
| Somenek, 2024 (12) | Serious | Low | Low | Low | Low | Serious | Low |
| Bravo, 2022 (9) | Serious | Low | Low | Low | Low | Low | Low |
| Zerbinati, 2023 (33) | Serious | Low | Low | Low | Low | Serious | Low |
| Kadough, 2022 (11) | Serious | Low | Serious | Serious | Low | Serious | Low |
| Wortsman, 2023 (30) | Serious | Low | Serious | Serious | Low | Low | Low |
| Godin, 2006 (15) | Serious | Low | Serious | *No information* | Serious | Serious | Low |
